# Radiogenomics analysis identifies correlations of digital mammography with clinical molecular signatures in breast cancer

**DOI:** 10.1371/journal.pone.0193871

**Published:** 2018-03-29

**Authors:** Jose-Gerardo Tamez-Peña, Juan-Andrés Rodriguez-Rojas, Hugo Gomez-Rueda, Jose-Maria Celaya-Padilla, Roxana-Alicia Rivera-Prieto, Rebeca Palacios-Corona, Margarita Garza-Montemayor, Servando Cardona-Huerta, Victor Treviño

**Affiliations:** 1 Cátedra en Bioinformática, Escuela de Medicina, Tecnológico de Monterrey, Av. Morones Prieto Pte 3000, Col. Los Doctores, Monterrey, NL, México; 2 Universidad Autónoma de Zacatecas, Zacatecas, Zacatecas, Mexico, CP; 3 Centro de Investigación Biomédica del Noreste, Instituto Mexicano del Seguro Social Ave. 2 de Abril y San Luis Potosí s/n, Col. Independencia, Monterrey, NL, México, CP; 4 Centro de Cáncer de Mama, Hospital Zambrano Hellion, Av, Col. Real San Agustin, San Pedro Garza Garcia, Nuevo León, México; Tata Memorial Centre, INDIA

## Abstract

In breast cancer, well-known gene expression subtypes have been related to a specific clinical outcome. However, their impact on the breast tissue phenotype has been poorly studied. Here, we investigate the association of imaging data of tumors to gene expression signatures from 71 patients with breast cancer that underwent pre-treatment digital mammograms and tumor biopsies. From digital mammograms, a semi-automated radiogenomics analysis generated 1,078 features describing the shape, signal distribution, and texture of tumors along their contralateral image used as control. From tumor biopsy, we estimated the OncotypeDX and PAM50 recurrence scores using gene expression microarrays. Then, we used multivariate analysis under stringent cross-validation to train models predicting recurrence scores. Few univariate features reached Spearman correlation coefficients above 0.4. Nevertheless, multivariate analysis yielded significantly correlated models for both signatures (correlation of OncotypeDX = 0.49 ± 0.07 and PAM50 = 0.32 ± 0.10 in stringent cross-validation and OncotypeDX = 0.83 and PAM50 = 0.78 for a unique model). Equivalent models trained from the unaffected contralateral breast were not correlated suggesting that the image signatures were tumor-specific and that overfitting was not a considerable issue. We also noted that models were improved by combining clinical information (triple negative status and progesterone receptor). The models used mostly wavelets and fractal features suggesting their importance to capture tumor information. Our results suggest that molecular-based recurrence risk and breast cancer subtypes have observable radiographic phenotypes. To our knowledge, this is the first study associating mammographic information to gene expression recurrence signatures.

## Introduction

Breast cancer is the most frequent cancer among women worldwide [1]. The tumor grade, receptor status, and other characteristics are relevant for therapy selection and estimation of recurrence and survival [2]. Studies have found molecular heterogeneity suggesting the existence of intrinsic subtypes of breast cancer [3], and similar heterogeneity has been observed in imaging studies [3–7].

Gene expression analysis of breast cancer has led to several signatures, but only a few of them are used in a clinical setting [8]. The Food and Drug Administration has approved the commercialization of some signatures [9–11]. The OncotypeDX signature, based on 21 genes, was originally used to stratify estrogen receptor (ER) positive, lymph node (LN) negative, HER-2 neu normal breast cancer into recurrence risk categories [10]. Recently, this tool has also been used in lymph-node positive breast cancer [12]. The PAM50 is a 50-gene based signature that classifies tumors into five subtypes providing prognostic information for untreated and tamoxifen-treated patients [11–13].

The association between molecular properties of tumors and their image information (image phenotype) has not been widely studied. In this context, radiomics refers to an emerging methodology of extracting large amounts of features from medical images including intensities, shape, texture among many others [5,14–19], and radiogenomics is referred as the methods to associate genetic information with medical images [19–22]. Recent radiogenomics studies have found that molecular markers and clinical variables of interest correlate with magnetic resonance imaging (MRI) features of tumors in breast, kidney, lung, and brain cancer [14,23–29]. The evidence suggests that the activity regulated by gene expression leads to the development of architectural patterns that can be identified using non-invasive imaging. However, the resolution of MRI is low compared to mammography possibly obscuring fine details. Moreover, MRI is expensive to be used routinely in clinics.

Among the breast imaging modalities, mammography is the recommended modality for routine breast-cancer screening [30]. Mammograms are often the first evidence of the existence of a tumor, providing information about the anatomical location and extent of the tumor. Additionally, mammograms are commonly available, relatively cheap, and more operator-independent than ultrasound imaging. However, the relationship between mammographic features and gene expression patterns has not been widely examined.

The aim of this study was to investigate the association between imaging tumor phenotype obtained from digital mammograms and breast cancer gene expression signatures, particularly those from OncotypeDX and PAM50. We analyzed 71 breast cancer patients where mammogram and biopsy were acquired before surgery and systemic treatment. To our knowledge, this is the first dataset containing these data. Using a highly stringent cross validation strategy, we first show that image from tumor data is able to significantly predict molecular recurrence scores. As a negative control, the prediction from data of the contralateral breast not containing tumor is significantly lower indicating, as expected, that prediction is more informative from tumor data. We then analyzed possible recurrence biomarkers from image data and found levels of correlations that could be utilized in clinics. From the features selected in putative image biomarkers, most are derived from wavelets and fractals suggesting that these types of information need to be further explored to find biological relevance. We also found that clinical indicators improve the predictions of image biomarkers. We conclude that digital mammography can be used to predict recurrence.

## Results

**Fig 1:**
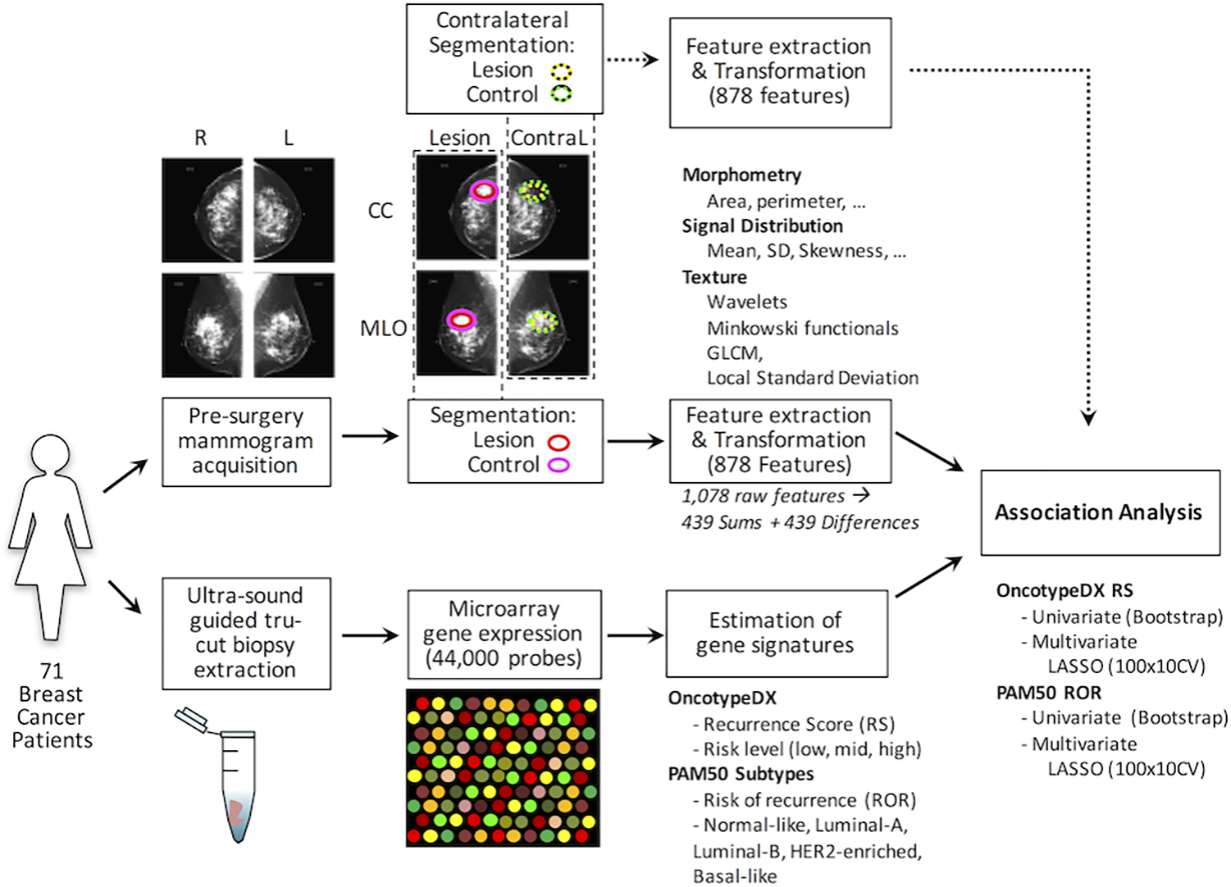
Radiogenomics pipeline used in the analysis of association between imaging features and gene signatures in patients with breast cancer. First, mammograms and tumor biopsy samples were acquired before surgery or treatment. A trained radiologist delimited the lesion region of interest to calculate Image features. For tumor biopsy samples, RNA was extracted and gene expression was measured using microarray technology, then the PAM50 molecular subtype and OncotypeDX recurrence score were measured. Univariate association based on correlation was used to show that image features are associated to signatures. Multivariate analysis was used to fit predictive models using cross-validation strategies and a feature selection algorithm. A similar procedure was used for contralateral images to evaluate whether the associations were tumor-specific.

A schematic view of the analysis pipeline is depicted in Fig 1. Briefly, we acquired routine four-image digital mammograms and core biopsy from 71 breast cancer patients (Table 1 and S1 Table). The mammography was computationally analyzed to extract 1,078 features from each region of interest (ROI), summarized in Table 2. On the mammogram obtained from the affected side, the original ROI was marked by the radiologist (MGM), surrounding the tumor and was processed to select the most informative ROI. A similar sized ROI from an apparent normal region of the mammography was used to estimate background feature values (S1 Fig and Methods section). The raw data is deposited in S2 Table for the lesion and S3 Table for the background-control. The data from Medio-lateral-oblique (MLO) and Cefalo-Caudal (CC) views were then condensed into sums or differences to finally generate 878 summary features (S4 Table, see the Methods section for details). As a negative control of the procedure, from the contralateral side mammogram not containing tumor, a ROI in an equivalent mirror position to the tumor and their corresponding background ROI were also obtained and analyzed (S5, S6 and S7 Tables). The biopsies were used to perform microarrays obtaining 44,000 gene expression features (S1 File), which were employed to estimate two clinical signatures of recurrence (OncotypeDX and PAM50 risk subtypes based on risk of recurrence scores). The image features were then used to search for associations with clinical signatures and gene expression features. The details are described in the Method section.

**Table 1:** Summary of patients per risk group.

	Total	Low Risk: 10 Normal-like, 11 LumA	High Risk: 22 LumB, 11 HER2+, 17 Basal-like	p for Difference
**No. of patients**	71 (100)	21 (29.5)	50 (70.4)	0.0004
**Avg. age**	50.8	51.5	50.5	0.7095
**Age < 50**	33 (46)	9 (42.8)	24 (48.0)	0.7965
**Menopausal status**				0.7965
**Pre-menopausal**	33 (47.8)	9 (45.0)	24 (49.0)	
**Post-menopausal**	36 (52.2)	11 (55.0)	25 (51.0)	
**Histological grade**				1.0000
**1-Well differentiated**	6 (8.5)	2 (9.5)	4 (8.0)	
**2-Moderatly diff.**	37 (52.1)	11 (52.4)	26 (52.0)	
**3-Poorly diff.**	23 (32.4)	7 (33.3)	16 (32.0)	
**NA**	5 (7.0)	1 (4.8)	4 (8.0)	
**Tumor size**				0.3280
**T1 (< = 20 mm)**	8 (11.3)	4 (19.1)	4 (8.0)	
**T2 (21–50 mm)**	44 (62.0)	12 (57.1)	32 (64.0)	
**T3 (>50 mm)**	18 (26.8)	4 (19.1)	14 (28.0)	
**Node status**				1.0000
**N0**	13 (18.3)	4 (19.0)	9 (18.0)	
**N1**	31 (43.7)	9 (42.6)	22 (44.0)	
**N2**	24 (33.8)	7 (33.3)	17 (34.0)	
**N3**	2 (2.8)	0	2 (4.0)	
**Metastasis status**				1.0000*
**M0**	63 (88.7)	19 (90.5)	44 (88.0)	
**M1**	5 (7.0)	1 (4.8)	4 (8.0)	
**Mx**	3 (4.2)	0	2 (4.0)	
**Immunohistochemical**				0.0169*
**HR+ (ER / PR), HER2-**	35 (49.3)	13 (61.9)	22 (44.0)	
**HR- (ER & PR), HER2+**	13 (18.3)	6 (28.6)	7 (14.0)	
**HR+ (ER / PR), HER2+**	5 (7.0)	0	5 (10.0)	
**HR- (ER & PR), HER2- (TN)**	17 (23.9)	1 (4.8)	16 (32.0)	
**(NA)**	1 (1.4)	1 (4.8)	0	

For patient counts, numbers in parenthesis represent percentages. LumA = luminal A, LumB = luminal B, HR = Hormone Receptors, ER = Estrogen Receptor, PR = Progesterone Receptor, TN = Triple Negative. Numbers represent only those patients with available information.

* Removing (NA) or Mx.

**Table 2:** Summary of the 539 features obtained from each region of the mammogram.

Group	Features	Description
**Signal distribution**	25	Describe the pixel-wise variations of the ROI signal intensities. Particular emphasis was added in the smaller and larger quantiles.
**Fractals**	25	A set of texture features based on signal distribution of the local fractal dimension.
**Gray Level Co-Occurrence**	24	Haralick’s grey-level co-occurrence matrix texture features: energy, entropy, correlation, difference moment, inertia, cluster shade, cluster prominence, and Haralick correlation at 3 distances (1, 2, and 4 pixels).
**Minkowski functionals**	40	Ten values (curvature energy, kurtosis, largest derivate, mean, half of maximum height, skewness, energy of the derivate, order of largest derivate, signal standard deviation, functional standard deviation) of the area, contour length, Euler number, and compactness of the image resulting from the binarization of the ROI at 40 uniformly spaced threshold values.
**Wavelet decompositions**	400	Decomposition of the images using the Daubechies-4 mother wavelet in 2D at 4 multi-resolution levels. The resulting images were subject to the signal distribution and fractal feature extraction process (4x4x25).
**Local standard deviation**	25	Signal distribution of the standard deviation from a 3x3 pixel image.

### Cancer subtype and recurrence risk estimation from gene expression signatures

We estimated the OncotypeDX risk score (OncotypeDX RS) and the PAM50 risk subtype from its risk of recurrence score (PAM50 ROR) from the microarray gene expression measurements (S1 Table) similarly to original publications. From OncotypeDX RS we observed 20, 4, and 47 patients for low, intermediate, and high-risk respectively according to the threshold values used in the original assays. From PAM50, 10 patients were identified as Normal-like, 11 as Luminal-A, 22 as Luminal-B, 11 as HER2 enriched, and 17 as Basal-like. These subtypes correspond to 21 low-risk (Normal-like and Luminal-A) and 50 high-risk (Luminal-B, HER2, and Basal-like). The overall numbers of low- and high-risk assignments from both signatures are similar to those expected given the TNM and immunohistochemistry results (Table 1). Comparing the discrete scores from both signatures (S2 Fig), the patients with low or medium OncotypeDX RS were in substantial agreement with Normal-like or Luminal-A subtypes (Cohen’s kappa: k = 0.76, p<0.05). As reported [31,32], the Basal-like subtype was enriched in triple-negative breast cancers (14 of 17). Moreover, the continuous recurrence score from both signatures were highly correlated (Spearman *ρ* = 0.86, p<2e-16). Hence, to avoid highly redundant information for association with image features, we used the score from OncotypeDX RS and the risk subtypes given by the PAM50 ROR score. Overall, these findings are consistent with previous reports [33,34] supporting the validity of our gene expression assays and our PAM50 and OncotypeDX estimations. Therefore, these estimations can be used as sensible effective surrogates of risk of recurrence.

### Image-based features are associated to OncotypeDX recurrence score

We used univariate and multivariate procedures to explore the association between image features and gene expression-based risk of recurrence from our estimations of OncotypeDX. We tested the association of individual image features with OncotypeDX RS using Spearman regression in a robust manner using bootstrapping. We observed that imaging features alone had moderate Spearman correlation, only 6 features whose correlation was |*ρ|* > 0.4 and 29 features at the |*ρ*| > 0.3 level (S8 Table). These features were majorly derived from fractals and wavelets. From the top 50 features, in general, we observed positive correlations to fractals and negative correlations for wavelets (S3 Fig). These results suggest that there are considerable correlations in the image data but also that the prediction of OncotypeDX RS from a unique image feature is limited.

**Fig 2:**
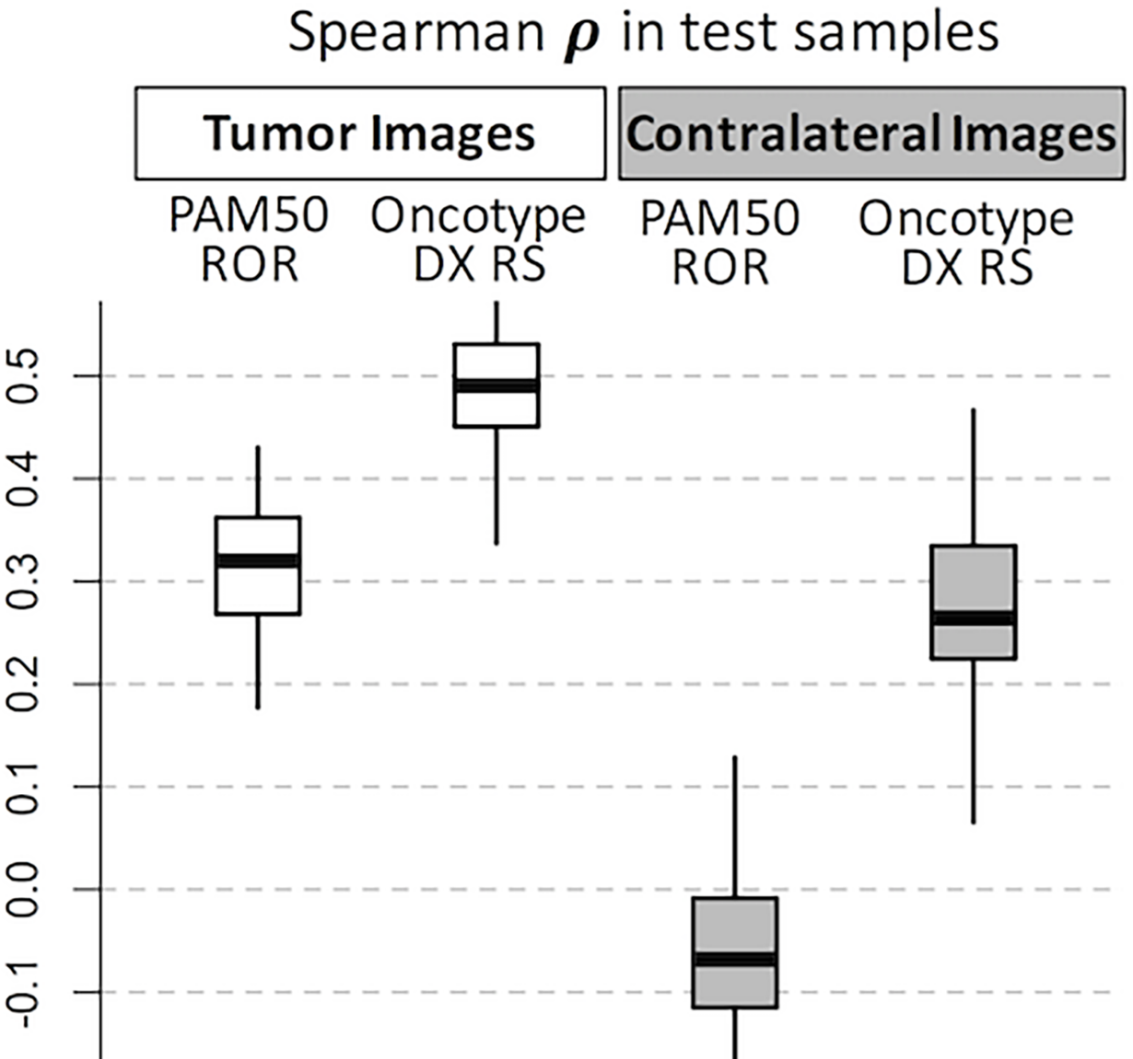
Distribution of test correlations in the cross-validation multivariate feature selection. White boxplots correspond to tumor ROIs whereas grayed boxplots correspond to contralateral ROIs used as controls. Note that contralateral distributions of test predictions are lower than corresponding tumor test predictions.

To improve on the univariate prediction and to potentially generate a multivariate signature, we evaluated whether multivariate signatures can generate confident predictions from our data and sample size. For this, we used 100 repetitions of a 10-fold-CV procedure using LASSO (see S4 Fig and the Methods section for details). We evaluated the prediction over the blind test set of the models and the stability of its included features counting the number of times each individual feature was obtained within the 1000 models. The median of Spearman correlations from the 100 test estimations was *ρ* = 0.49 [IQR 0.41–0.57] (Fig 2). These results indicate an improvement over the univariate correlation in a more realistic scenario. From the 1000 models (100 rounds of 10-Fold-CV) the top 5 most frequent features (S9 Table) were ranked 5^th^, 44^th^, 23^rd^, 6^th^, and 82^nd^ according to the univariate evaluation. This indicates that most frequent features in the multivariate are not necessarily top ranked in the univariate procedure.

Furthermore, we tested whether the OncotypeDX RS prediction is more favorable for tumor image data, thus we applied the same procedure of training and test from contralateral images. This correspond to the assumption that the unaffected contralateral data should provide scarce prediction because of the lack of apparent tumors. We noted that the median of Spearman correlations from the 100 test estimations in the contralateral data was low reaching only *ρ* = 0.26 [IQR 0.16–0.37] compared to *ρ* = 0.49 from the tumor image data (Fig 2) and most of the correlation estimations were not significant (S5 Fig). These results suggest that the image data is more correlated to OncotypeDX RS from the tumor ROI than to similar ROI from the unaffected contralateral tissue.

### Image-based features are associated with PAM50 subtypes

Similar to the procedure applied to OncotypeDX recurrence score, we also used the univariate and multivariate methods to investigate associations between the risk estimated by PAM50 and image-based features. Here, we expected similar results to those from OncotypeDX because the recurrence scores of PAM50 and OncotypeDX are correlated in our data. In the univariate analysis, we found 10 features whose univariate Spearman correlation was |*ρ*| > 0.4 and 48 features at the |*ρ*| > 0.3 level (S10 Table). Consistently to OncotypeDX, we also observed higher values of wavelets features in lower scores and higher values of fractals features to higher scores within the top 50 features (S5 Fig). In the multivariate analysis using 100 rounds of 10-fold-CV LASSO runs, we noted higher values of correlation using the tumor data (*ρ* = 0.32 [IQR 0.23–0.41]) than using the contralateral data (*ρ* = -0.06 [IQR -0.17–0.04]) (Fig 2).

### Assessing the prediction of image-based models for potential clinical use

**Fig 3:**
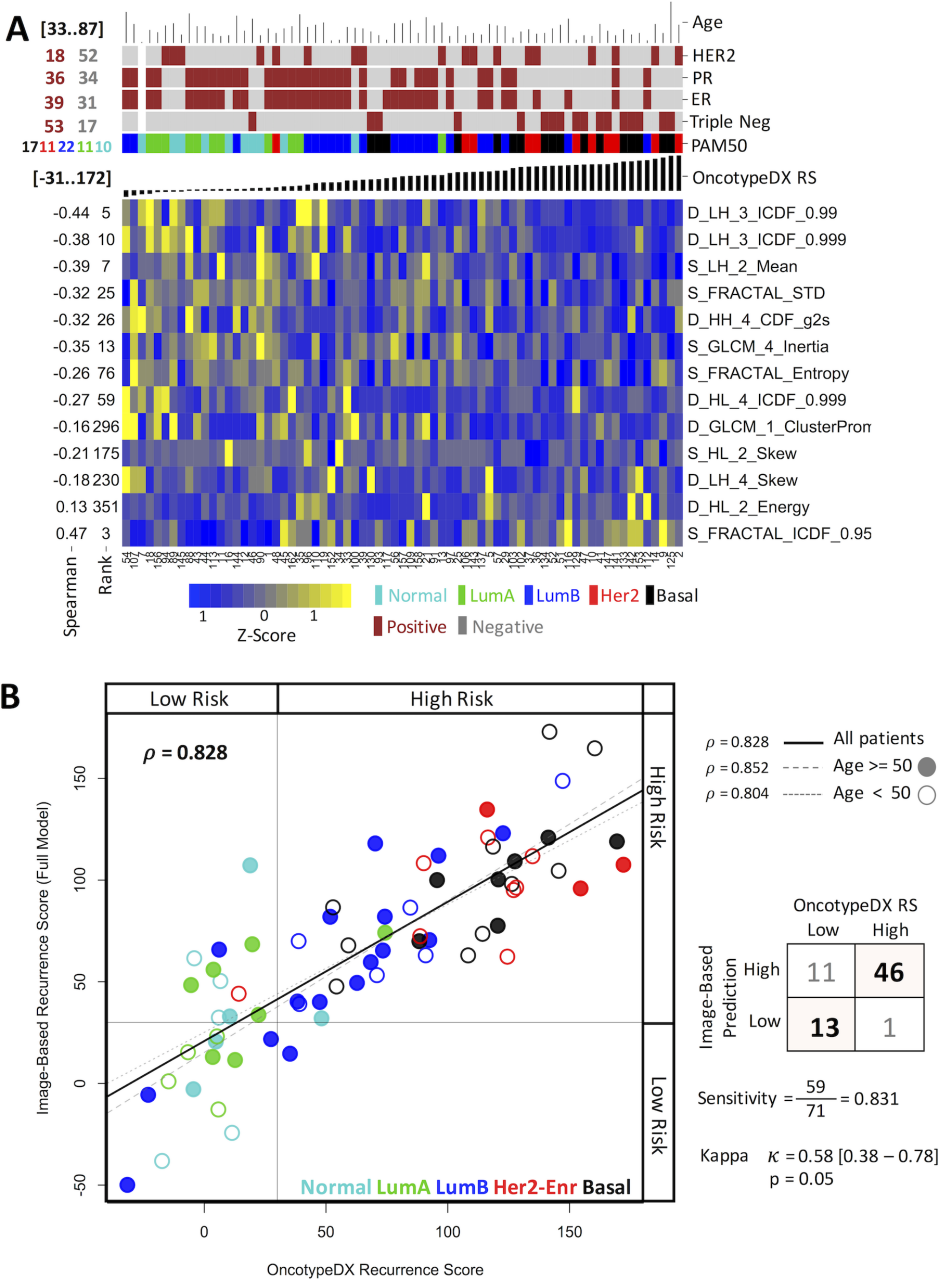
Characteristics of the model obtained for OncotypeDX. (A) A heat map representation of the features associated to OncotypeDX RS. The figure shows the features selected by LASSO (vertical axis) and their univariate Spearman coefficient and rank along samples (horizontal axis) ordered by the OncotypeDX risk score. The top of the figure includes common clinical indicators. The image data was scaled to z-score to nightlight differences. (B) Comparison of the estimated OncotypeDX recurrence score with that of the score predicted by the image model in (A). Each dot represents a sample. Colors represent subtypes and filled or open circles represent younger or older patients.

**Fig 4:**
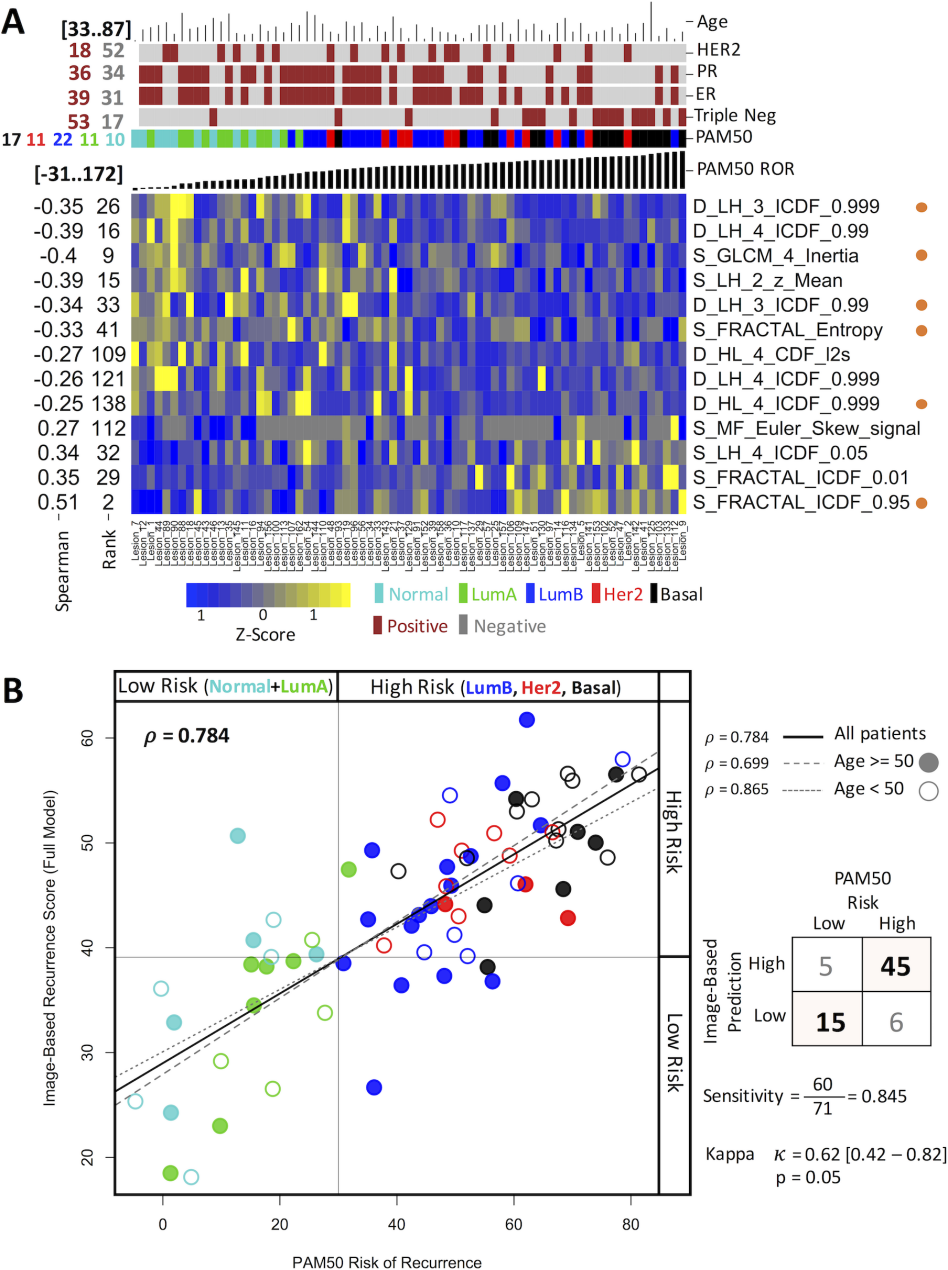
Characteristics of the model obtained for PAM50. (A) A heat map representation of the features associated to risk from PAM50 ROR. The figure shows the features selected by LASSO (vertical axis) and their univariate Spearman coefficient and rank along samples (horizontal axis) ordered by the PAM50 ROR score. The top of the figure includes common clinical indicators. The image data was scaled to z-score to nightlight differences. Orange dots at the right represent features also present in the OncotypeDX model. (B) Comparison of the estimated PAM50 recurrence score with that of the score predicted by the image model in (A). Each dot represents a sample. Colors represent subtypes and filled or open circles represent younger or older patients.

Once we showed that image features could be used to predict clinical relevant indicators and that these predictions are more powerful for the tumor ROI, we explored the possibility of providing a unique prediction for an unseen sample in a putative clinical setting. From all data, we generated a unique model using LASSO. The generated model for OncotypeDX RS consists of 13 image features including 8 from wavelets, 3 from fractals, and 2 from gray level co-occurrence matrix (Fig 3A). Using this model, the correlation of the estimated OncotypeDX RS and the image-based prognostic model is high (Fig 3B Pearson *ρ* = 0.828, p < 10^−15^) and the risk assignment provided by both indicators highly concurs (Kappa *k* = 0.58, p < 0.05). This correlation is conserved among younger (age < 50) or older (age > 50) patients. Correspondingly for PAM50, the model using LASSO and the 71 mammograms (Fig 4) highly correlates with the PAM50 risk score (*ρ* = 0.784, p < 10^−9^). For radiologists, it has been known that the molecular subtypes show some image properties [35]. Thus, because PAM50 subtypes and the risk of recurrence from PAM50 are highly correlated, we asked whether the model could be useful to classify into molecular subtypes. We estimated that, to classify subtypes into low-risk (Normal-Like or LuminalA) and high-risk (LuminalB, or Her2- enrich, or Basal-Like), the sensitivity was 84.5% and the similarity of the prediction was statistically significant (Kappa k = 0.62, p < 0.05).

### Image features related to molecular signatures

**Fig 5:**
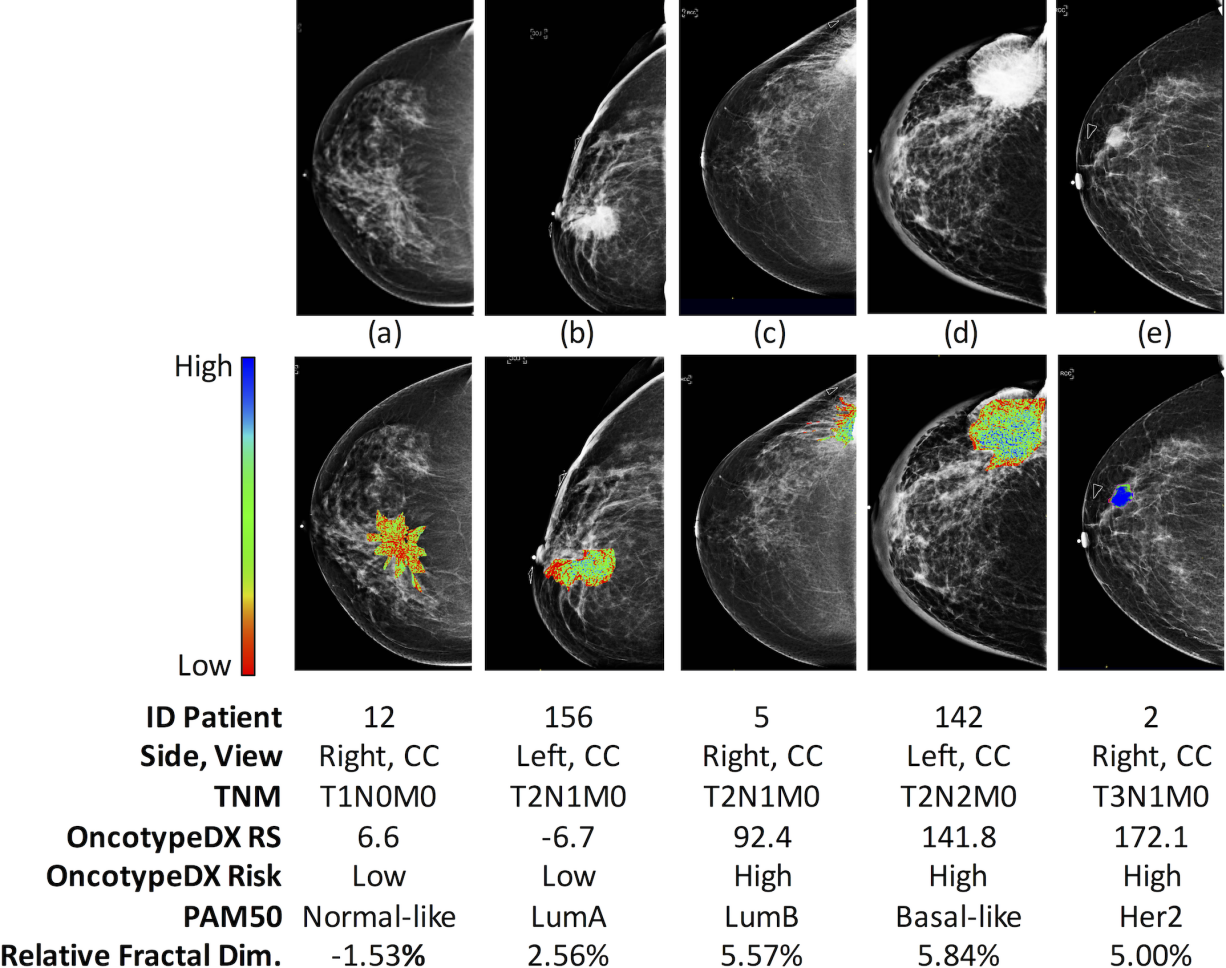
Color representation of the distribution of the local fractal dimension. High values indicate heterogeneous textures whereas low values represent uniform distributions of the tissue signal. Shades of blue, green, and red colors represent high, medium, and low dimension values respectively.

Of the top selected image features in OncotypeDX RS (Fig 3), 8 were from wavelets, 3 from fractals, and 2 from gray level co-occurrence matrix. Other image features were rarely included such as those grouped in signal distribution, Minkowski Functionals and local standard deviation. A similar result was observed from PAM50 where top features were obtained from wavelets and fractals (Fig 4). This implies that very specific sets of radiological textures of the tumors are different between low- and high-risk patients where wavelets and fractals stand out. We observed 6 features present in both predictors that could be due to the correlation between PAM50 ROR and OncotypeDX RS. In addition, to visually support the relationship of the image measurement to the tumor ROI, a graphical representation of a feature associated to fractals included in the LASSO model is shown in Fig 5.

### Clinical data improves associations to image-based features

The associations of image features to molecular signatures shown above were based on image features alone. However, in clinical practice some important markers and risk factors are available before or after surgery and routine pathological inspection and assays. Therefore, we tested whether age, TNM histological grades, estrogen, progesterone, HER2, and triple negative status can add information to predict PAM50 subtypes or OncotypeDX RS in the presence of image based data. For this, we also ran LASSO for all samples competing image and clinical information. The overall results show an increase in LASSO prediction models for OncotypeDX RS and PAM50. Interestingly, for OncotypeDX RS, we observed that the progesterone receptor and the triple negative status were used in the model. Whilst for PAM50, we observed only the triple negative status included in the model. We did not observe TNM, ER, HER2, or Age in models once image data was considered. These results suggest that image data can compete in prediction with clinical data and that both types of information are useful.

## Discussion

This radiogenomic study found that mammographic features were correlated to the breast cancer recurrence score provided by OncotypeDX and PAM50, and to the overall risk from breast cancer molecular subtypes. Predictive image-based models support these findings. This also indicates that the common mammography is able to capture molecular information. Notably, our mammography-based results are akin to other exploratory breast MRI-based studies [5,25,26]. The use of radiomic features from digital mammograms allows the use of low-cost and widely available technology to obtain surrogate measurements for validated gene signatures. To our knowledge, this is the first study showing such associations.

The association of image features to gene expression is rather challenging this can be partly explained by the fact that individual image features may be associated to complex combinations of genomic expressions. During the feature extraction process, we intended to capture several aspects of the signal distribution and parenchymal texture of the mammograms to increase the likelihood of correlation. Through multivariate analysis we were able to build mammography-based models with substantial agreement to validated gene signatures. Most of the considered aspects included in models suggest that tumor characterization requires multiple features representing different characteristics of the image. In addition, we observed that features in models were not necessarily the top ranked in a univariate analysis suggesting that a simple model combining top univariate features in not obvious supporting the use of more sophisticated methods to generate an image signature. Thus, a reasonable approach to capture tumor behavior complexity is through radiomic multivariate analysis. Nevertheless, multivariate analyses increase the space of solutions rising the risk of overfitting. To avoid this, we used 100 rounds of stringent cross-validations and also employed the contralateral images as a negative control.

The associations found of image features to molecular signatures were mainly based on texture features (fractals and wavelets) supporting the findings of previous CAD studies that have shown different patterns between normal breast tissue and tumors [36–41]. Although texture features using wavelets have been explored for breast cancer diagnosis [42,43], to our knowledge, this is the first time that wavelets features have been successfully used to predict recurrence risk. Furthermore this study support the reports that have associated image features with the risk of recurrence on ductal carcinoma in situ [44] and that molecular risk subtypes can be differentiated by imaging [45]. We used features composed of the sum of both views (CC+MLO) and their absolute difference (|CC-MLO|) after reducing background from the tumor ROI. Both types of features were found in most frequently selected features in both risk predictors. This implies that both views are important and that more complex relationships and transformations of the values from CC and MLO and novel features could improve the correlations.

The multivariate unique models predicting the OncotypeDX and risk from PAM50 ROR signatures shared some features (Figs 3 and 4). This can be due to the confirmed correlation of OncotypeDX RS and PAM50 ROR. Nevertheless, it may also suggest that these features are strongly associated to a specific molecular property of the sampled tumors as indicated in mammograms (Fig 5).

Remarkably, the performance achieved by the mammogram-based models is comparable to that found in breast MRI-based studies [26,27]. It is important to mention that in the construction of our models, we used a more stringent cross-validation technique, more diverse subtypes of breast cancer samples, and digital mammography, which can be more easily replicated.

Initially, we did not use known risk factors such as radiological interpretation of the images (BIRADS, tumor size, margin, morphology), pathological information (tumor stage, receptor status, margin status), or personal information (age, GAIL score, family breast cancer history) [2]. Surprisingly, image features were still preferred by the feature selection algorithm than clinical data. Nevertheless, we have shown that the inclusion of such features could improve the performance of the models suggesting the combination of both clinical data and radiomics as a possible future direction for clinical trials.

There is evidence that tumor subtypes may be influenced by a genetic background [46]. However, the better and significant performance of the models obtained from the lesion rather than those obtained from the unaffected breast hints to the presence of macroscopic phenotype changes that are associated to tumor development. Our evidence supports the hypothesis that tumor recurrence and subtype can only be predicted by the images of the tumor tissue.

We used measurements of an ROI enclosing the tumor discriminating between higher dense regions and likely unaffected background tissue. This demonstrated that the molecular information related to images is derived specifically from the tumor. Nevertheless, for clinical use, our experimental procedure has the disadvantage of the determination of the ROI ahead of the radiomic feature extraction process. It would be more interesting, but by far more challenging, to determine automatically the ROI or to use the whole image without a priori ROI segmentation. Emerging methods in this direction are promising [47–49]. We have shown that the contralateral ROI was barely or not correlated in the test sets to the recurrence scores. However, the predicted risk values from the contralateral ROI were not necessarily low. This is not a surprising finding as previous studies has shown that the non-affected contralateral breast may have some information regarding the cancer event [50–52]. Our image-based models can be applied assuming the ROI is cancerous. Nevertheless, once we have shown that image-based models are correlated with risk, a similar methodology can be easily expanded to first classify normal, benign, and cancerous images before using risk prediction based on image-based models.

The size of our sampled cohort, the use of a single mammography equipment, the advanced stage of some of the cancer patients, and the lack of similar studies limit the generalization of the results. The participating subjects represent breast cancer patients from northeastern Mexico. Nevertheless, we consider that a larger and more heterogeneous radiogenomics cohort using similar analyses should show comparable findings since our methodology was rigorous in the training and test scheme and in the comparisons with the contralateral ROI. In our study we used the scores of validated signatures as the targets instead of observed clinical outcomes. Survival analysis using the imaging features would have allowed the creation of an entirely radiomic-based signatures and the identification of imaging intrinsic phenotypes associated to clinical outcomes as suggested in recent studies [14,26]. We will follow-up our population for long-term outcomes including disease-free and overall survival. Additionally, we did not found similar studies to compare the models and our methodology. Nevertheless, The Cancer Genome Atlas (TCGA) consortium, specifically the TCGA Breast Phenotype Research Group, is starting to make similar studies [7]. So, it will be possible to compare our results with other studies in the coming years. Consequently, the results and the methodology shown here could be valuable.

## Conclusion

Our radiogenomic study provides supporting evidence that mammography is able to capture changes associated with intrinsic molecular characteristics in breast cancer. This is supported by predictive image-based models that had comparable behavior to validated gene signatures for breast cancer molecular subtypes and recurrence risk. The potential use of imaging surrogates is promising and can be easily be implemented in clinics. In low-income countries, it may have a profound impact where a risk of recurrence estimation based on molecular signatures is economically prohibitive. Nevertheless, additional analysis is required to determine robust image-based signatures that are useful in a broader clinical setting. Fortunately, other researchers such as TCGA that already obtained molecular estimations of risk or other outcomes could validate our analyses by recovering the routinely mammograms.

## Materials and methods

### Study population

The prospective study “Exploratory study for image-based biomarker discovery of breast cancer and its biological validation” (THSJ-BC) was approved by the institutional review board. We enrolled patients with breast cancer identified by mammography or by clinical inspection. Breast cancer was pathologically confirmed for all patients. For inclusion, the following criteria had to be met: consent form, bilateral mammograms (cranial caudal CC, mid-lateral oblique MLO), clinical information (age, TNM grading, ER, PR, and HER2 status), and high quality of the tumor sample for RNA, and microarray hybridization. Seventy-one patients met the criteria. All participants provided informed consent. Table 1 shows a summary of the clinical and pathological characteristics and S1 Table shows the details per donor. Images were reviewed by MGM and patients were diagnosed and treated by SCH.

### Biopsy and gene expression microarrays

Tumor samples were obtained from ultrasound-guided tru-cut biopsy and immersed into RNA Later (Thermo-Fisher Scientific, Waltham, MA, USA). The samples were stored at 4°C for less than 5 days then stored at -80°C until processing. The extraction of mRNA was performed according to the RNeasy Mini Kit (Qiagen, Venlo, Netherlands). RNA integrity was corroborated with Experion Automated Electrophoresis System (BioRad, Hercules, CA, USA). The gene expression was measured employing microarrays containing 44,000 probes (Agilent, Santa Clara, CA, USA) using standard provider protocols and analyzed using a GenePix 4000B scanner (Molecular Devices, Sunnyvale, CA, USA). The raw intensities were logarithm transformed and quantile-normalized.

### Gene signatures estimations

From microarray data, we estimated the diagnostic scores of two well-known breast cancer gene signatures, OncotypeDX and PAM50 [10]. The PAM50 signature uses fifty genes to determine the molecular subtype of the tumor: normal-like, luminal A, luminal B, HER2-enriched, and basal-like [11]. To estimate the subtypes, we used the implementation available at https://genome.unc.edu/pubsup/breastGEO/PAM50.zip and the corresponding original dataset for training [11]. To compare the PAM50 risk assessment to OncotypeDX scores we grouped subtypes following previous observations [53]. Briefly, patients from Normal-like and Luminal A subtypes were grouped into a “low-risk” group, and the Luminal-B, HER2+, and Basal-like patients into a “high-risk” group. Twenty-one patients were low risk and 50 high risk, respectively.

OncotypeDX is a validated assay that estimates a score proportional to the probability of breast cancer recurrence within 10 years after treatment [10]. Additionally, we confirmed such findings *in-silico* for several ER and LN heterogeneous populations using the SurvExpress tool [54]. This result is included in the supplementary material of SurvExpress. Consequently, we used the recurrence score (RS) from the OncotypeDX signature as an approximation of the recurrence risk in our population. OncotypeDX uses 16 genes (plus five genes for normalization) to estimate a continuous score truncated to ranges from 0 to 100 for low to high risk respectively, three risk categories are assigned accordingly [10]. We used the R-package *genefu* [55] to estimate the continuous non-truncated recurrence score and risk category from microarray data and avoid additional non-linearities produced by the truncation process. As suggested for PAM50, a low risk group was defined as scores lower than 18 (20 patients), intermediate risk as 18 to 30 (4 patients) and high risk higher than 30 (47 patients).

### Image acquisition and feature extraction

2D Standard mammogram projections (CC and MLO) were acquired using a digital mammography system with automatic intensity adjustment (Selenia, Hologic, Bedford, MA) recorded at 70 microns per pixel and a grayscale of 12-bits. All mammograms images were evaluated by an experienced breast radiologist (MGM) who reported the relevant findings [56] and delineated the extension of the tumor using a computational tool (CiPAS, IMITEK, Monterrey, Mexico). All the segmentations were reviewed and manually refined to avoid the inclusion of pectoral muscle, skin folds or artificial markers. When two lesions were present in an image, only the lesion from which the tissue sample was extracted was taken into consideration. The segmented region of each image was processed to select the most informative region by image processing procedures determining the tumor region of interest (ROI) and the background ROI (S1 Fig). Both ROIs were used for the feature extraction procedure. For this, from each ROI, a set of radiomic features describing the shape, signal distribution, and texture of the tumor were calculated using a computational tool (CiPAS, IMITEK Mexico) and libraries from the Insight Toolkit (ITK) project (Kitware, Clifton Park, NY, USA). All computations were done using the DICOM stored images without any further post-processing. Most of the selected features of this analysis have already been shown to have some association with breast cancer risk [36,41,42,50,57–59]. Some other of the features were previously used for the prediction of the BIRADS score [60], and other features were included to explore their relevance in this application. Briefly, shape features described the area, contour and compactness from the borders of the ROI. Signal distribution features include descriptive statistical properties (i.e. signal mean, median, skewness, kurtosis), and intensities values at prescribed percentiles. Parenchymal texture was described using fractal dimension [41], gray level co-occurrence matrix (GLCM) features, Daubechies-4 wavelets features at four resolution levels, local standard deviation, and Minkowski Functionals (MF) summary features [61].

From each of these ROI, we extracted 539 unique features (Table 2) generating a total of 1,078 image features per tumor and 1,078 for the background (S2 and S3 Tables respectively). Because the mammographic presentation of the breast tissue varies among women, the background ROI was used to adjust for those differences. Then, to analyze the information from both views, we used the sum and absolute difference between CC and MLO views. We removed wavelets LL data because it represents the same information as the original image in lower resolution and to avoid problems of high feature correlations in the feature selection method. Finally, the data used as the input dataset consist of 878 image features corresponding to 439 sums and 439 differences (S4 Table).

To determine whether an image signature was tumor-specific, the tumor-free contralateral breast image was used as a control. The control-image features were manually defined in a similar location than the breast tumor. The equivalent image processing procedure was performed to tumor ROI in these contralateral images (S5 and S6 Tables for raw data and S7 Table for sums and differences from the 64 patients having complete contralateral information). In this set of data, we removed 5 patients having tumors in both breasts and 2 patients missing the contralateral mammography.

### Association analysis

The univariate association of individual imaging features with the OncotypeDX RS was measured using the Spearman correlation coefficient estimated by 100 random sample selections (bootstraps) of 90% of the samples similar to a 10-fold cross validation. To find multivariate models associating image features with OncotypeDX RS, we performed 100 repetitions of a feature selection method executed within a 10-fold-cross-validation procedure providing a robust and reliable estimate of the error in a blind test set independently of samples used for training (S2 Fig). For multivariate feature selection, we used LASSO [62]. LASSO performs a multivariate regression (over the OncotypeDX RS or the binary risk from PAM50 ROR) penalizing coefficients by a threshold value. Only the highest coefficients larger than the minimum error threshold were used for the regression. LASSO explores several values of the threshold value examining the error in each explored value. We used the cross-validated procedure from the LASSO algorithm implemented in the *glmnet* package in R. All model estimations and predictions were done using the default *glmnet* cross validation parameters with minimum error. Once the procedure to generate models was validated, we used all the samples to generate a LASSO model that could optimistically predict future mammographies.

## Supporting information

S1 Table Clinical data. Relevant clinical information of all individuals included in this study.
(XLS)

S2 Table Tumor raw image features. Raw image features obtained from the ROI of the tumor.
(XLS)

S3 Table Tumor raw image background features. Raw image features obtained from the ROI surrounding the tumor.
(XLS)

S4 Table Tumor image features sums-differences. Sums and Differences of the image features from the tumor.
(XLS)

S5 Table Contralateral raw image features. Raw image features obtained from the ROI of the contralateral not containing tumor.
(XLS)

S6 Table Contralateral raw Image background features. Raw image features obtained from the ROI surrounding the contralateral ROI.
(XLS)

S7 Table Contralateral image features sums-differences. Sums and Differences of the image features from the contralateral.
(XLS)

S8 Table Univariate correlation of image features to OncotypeDX. Bootstrapping estimations of the image features associated to OncortypeDX risk score.
(XLS)

S9 Table Frequency of features in 1000 models of OncotypeDX associations. Contains the frequency of features included in models from the 100 round of 10-Fold-CV for OncotypeDX risk score.
(XLS)

S10 Table Univariate correlation of image features to PAM50. Bootstrapping estimations of the image features associated to the risk subtype from the PAM50 ROR.
(XLS)

S1 Fig Image processing procedure. This figure shows the computational procedure followed to generate the region of interests (ROI) of the lesion and background from the radiologist’s trace.
(TIFF)

S2 Fig Correlation between the risk estimations of PAM50 and OncotypeDX. The Pearson correlation is shown. The estimated were also classified into low- and high-risk and compared using the kappa statistic.
(TIFF)

S3 Fig Top 50 image features associated to OncotypeDX by univariate estimation. The heat map shows the image features in vertical and the patients in horizontal. Data shown in in z-scores. The blue shades indicate low z-scores values whereas yellow indicates high z-scores values. The univariate Spearman correlation coefficient and the rank per image feature is shown. Patients were sorted according to the OncotypeDX estimation. The PAM50 subtype is shown at top for comparison.
(TIFF)

S4 Fig Process to generate cross-validated models using LASSO. (TIFF)

S5 Fig Top 50 image features associated to PAM50 by univariate estimation. The heat map shows the image features in vertical and the patients in horizontal. Data shown in in z- scores. The blue shades indicate low z-scores values whereas yellow indicates high z-scores values. The univariate Spearman correlation coefficient and the rank per image feature is shown. Patients were sorted according to the PAM50 estimation. The PAM50 subtype is shown at top.
(TIFF)

S1 File Microarray gene expression data. Compressed file containing the log-transform gene expression data.
(ZIP)
