# Supplementary figures and images for: Radiogenomics analysis identifies correlations of digital mammography with clinical molecular signatures in breast cancer
Source: PLoS One. 2018 Mar 29;13(3):e0193871. doi: 10.1371/journal.pone.0193871 (PMC5875760; doi:10.1371/journal.pone.0193871)

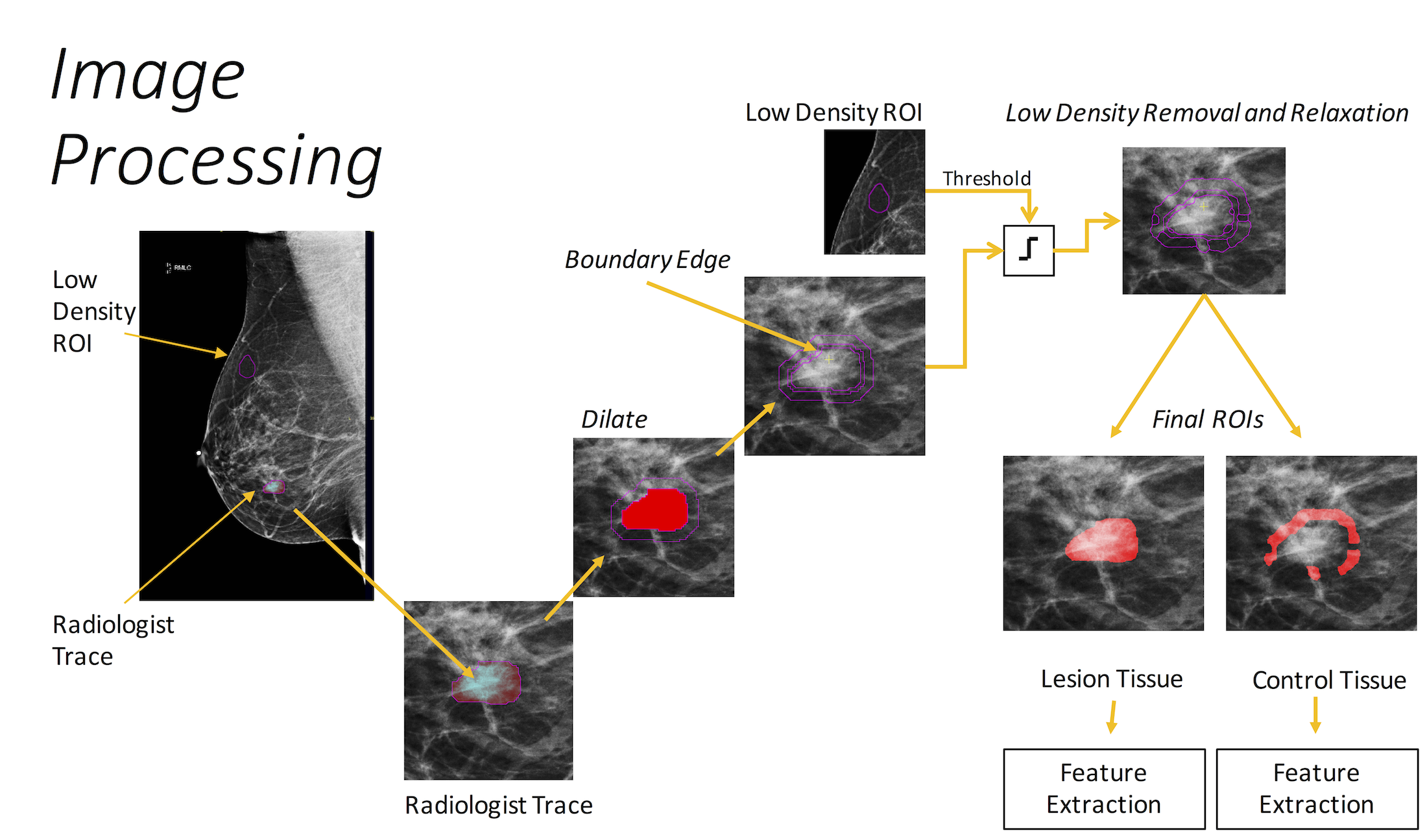

Supplement: S1 Fig — This figure shows the computational procedure followed to generate the region of interests (ROI) of the lesion and background from the radiologist’s trace. (TIFF) [file pone.0193871.s011.tiff]

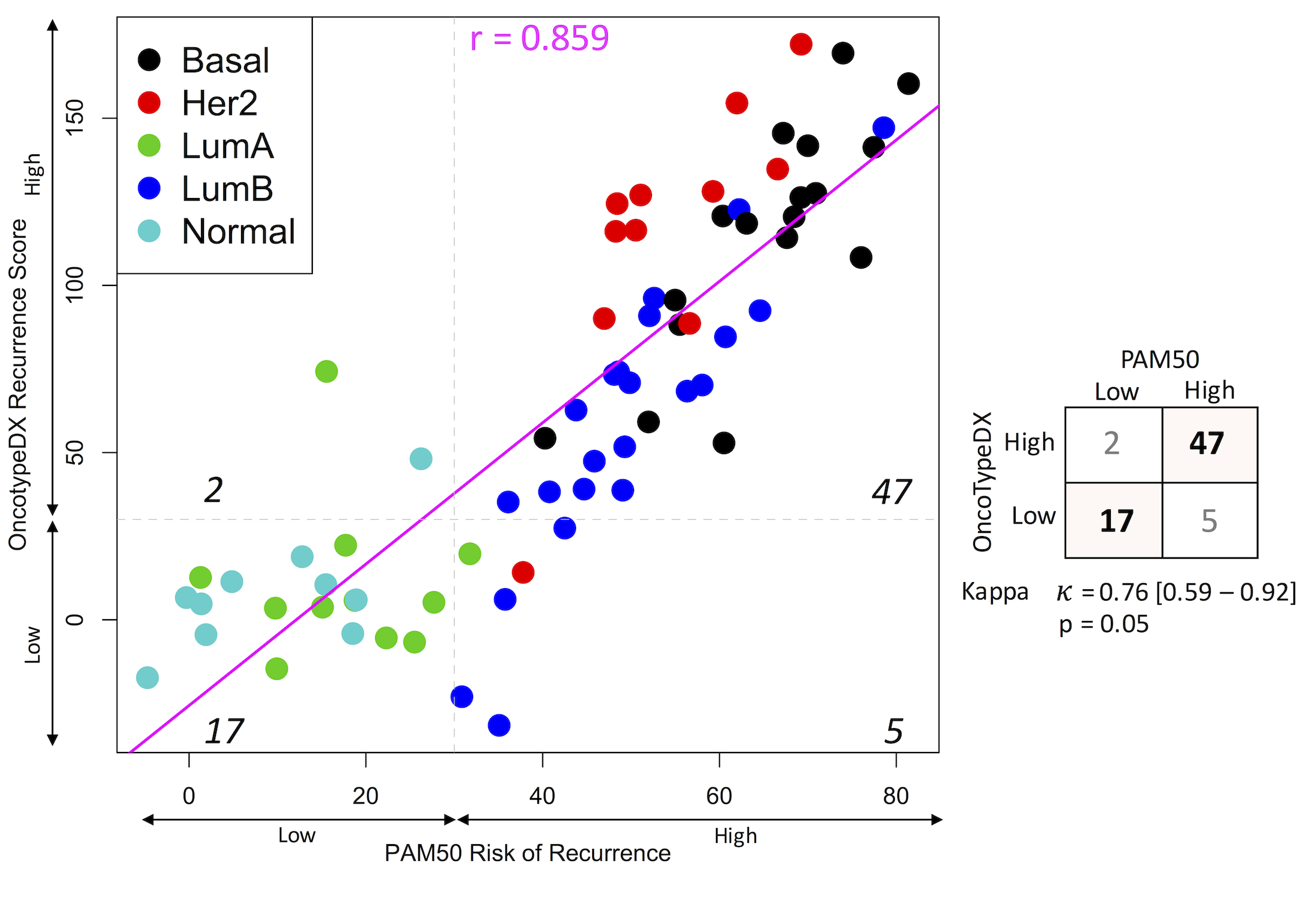

Supplement: S2 Fig — The Pearson correlation is shown. The estimated were also classified into low- and high-risk and compared using the kappa statistic. (TIFF) [file pone.0193871.s012.tiff]

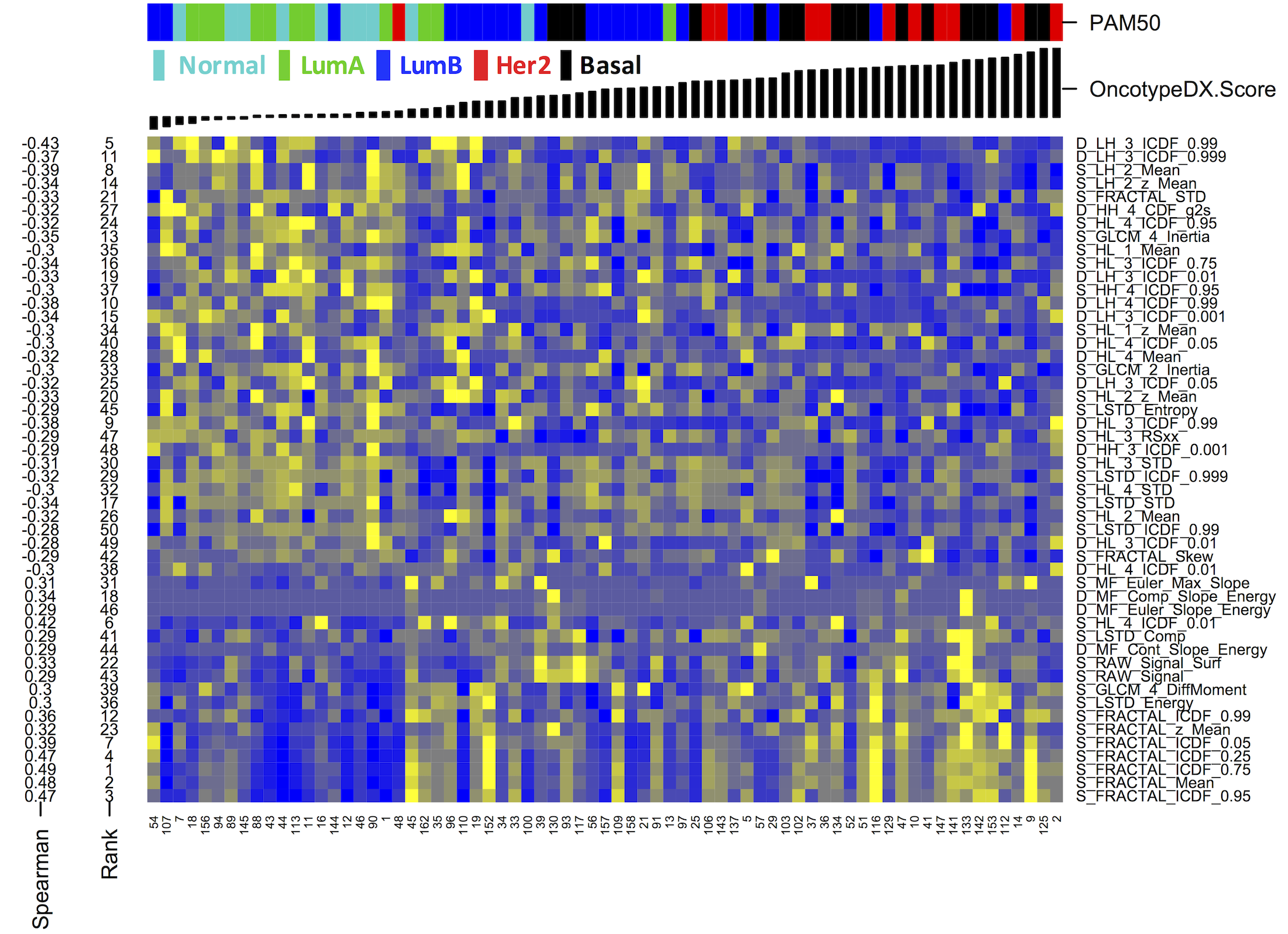

Supplement: S3 Fig — The heat map shows the image features in vertical and the patients in horizontal. Data shown in in z-scores. The blue shades indicate low z-scores values whereas yellow indicates high z-scores values. The univariate Spearman correlation coefficient and the rank per image feature is shown. Patients were sorted according to the OncotypeDX estimation. The PAM50 subtype is shown at top for comparison. (TIFF) [file pone.0193871.s013.tiff]

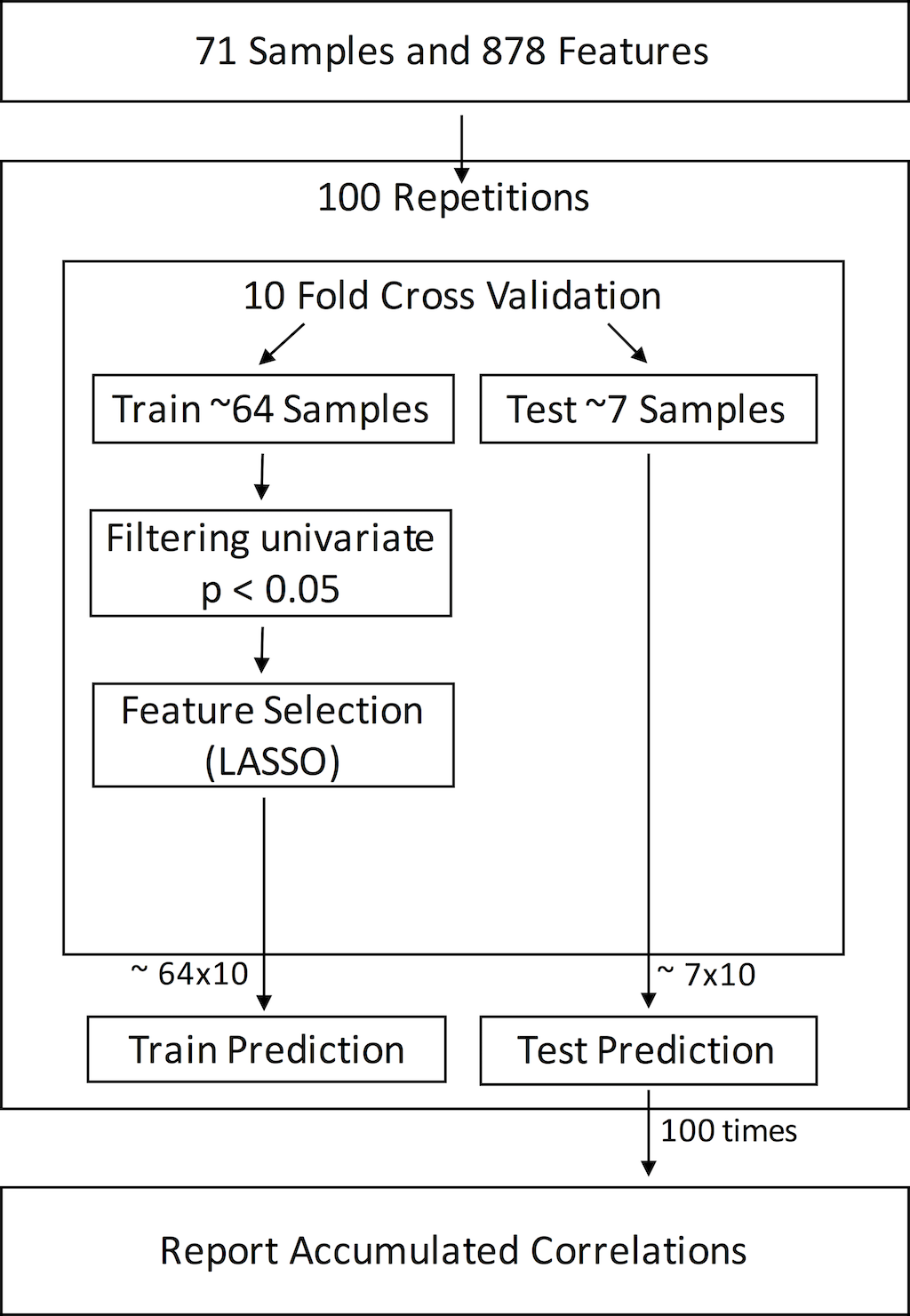

Supplement: S4 Fig — (TIFF) [file pone.0193871.s014.tiff]

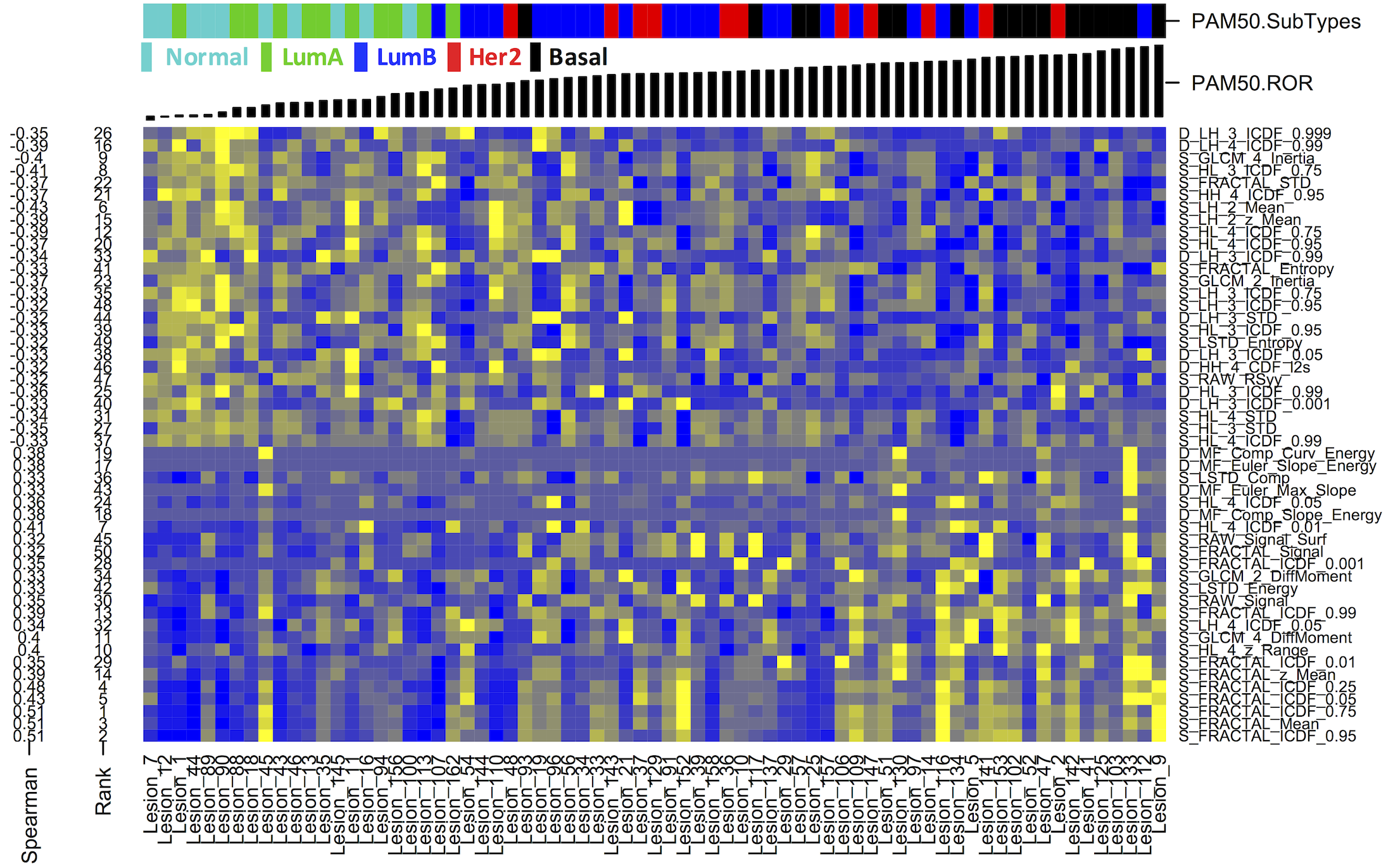

Supplement: S5 Fig — The heat map shows the image features in vertical and the patients in horizontal. Data shown in in z- scores. The blue shades indicate low z-scores values whereas yellow indicates high z-scores values. The univariate Spearman correlation coefficient and the rank per image feature is shown. Patients were sorted according to the PAM50 estimation. The PAM50 subtype is shown at top. (TIFF) [file pone.0193871.s015.tiff]
